# What does it take to support community-engaged research in an academic medical center? A mixed-methods study of community and academic perspectives

**DOI:** 10.1017/cts.2026.10771

**Published:** 2026-07-16

**Authors:** Prajakta Adsul, Alena Kuhlemeier, Mark Wieland, Irene Sia, Sagar Dugani, Vesna D. Garovic, Nina Wallerstein

**Affiliations:** 1 Division of Epidemiology, Biostatistics, and Preventive Medicine, Department of Internal Medicine, School of Medicine, University of New Mexico Health Sciences Center, Albuquerque, NM, USA; 2 Cancer Control and Population Sciences Research Program, Comprehensive Cancer Center, University of New Mexico, Albuquerque, NM, USA; 3 Center for Advancing Dissemination and Implementation Science, University of New Mexico Health Sciences Center, Albuquerque, NM, USA; 4 University of New Mexico Health Sciences Center, Albuquerque, NM, USA; 5 Community Internal Medicine, Mayo Clinic, Rochester, MN, USA; 6 Mayo Clinic, Rochester, MN, USA; 7 College of Population Health, University of New Mexico Health Sciences Center, Albuquerque, NM, USA

**Keywords:** Community-based participatory research, institutional context, community-engaged research, contextual assessment, institutional trustworthiness

## Abstract

**Introduction:**

There is growing focus on community-engaged research (CEnR) in the clinical and translational sciences, supportive policies, environments, and processes within academic medical centers. The purpose of this evaluation was to gather academic and community member perspectives on key actionable strategies for supporting CEnR in an academic medical center.

**Materials and Methods:**

We used a sequential mixed method design by first conducting qualitative in-depth interviews with academic leaders, investigators, and staff (total, *n* = 54) and focus group discussions with community members (*n* = 33). Themes from these qualitative data informed the adaptation of an institutional survey that gathered perspectives from a broader group of academic and community members affiliated with the institution (*n* = 85). Both qualitative and quantitative data were integrated to inform actionable strategies.

**Results:**

Survey findings revealed that the institution was perceived as committed to health equity and CEnR, but institutional respondents expressed more concerns than community members about instrumental support of the work. Interviews and focus groups highlighted the opportunity to better align research efforts with community needs through more robust CEnR infrastructure and through interface with the clinical practice to promote patient access. Academic and community members emphasized the need for authentic, sustained engagement beyond federal mandates, including financial investment, capacity-building, and fostering trust. Leaders acknowledged structural challenges, siloed efforts, and the necessity for stronger coordination to increase integration of community engagement into the institutional mission.

**Conclusion:**

Findings suggest that aligning academic leaders, researchers, staff perspectives, and community members is essential to support and promote CEnR at an institutional level.

## Background

Community-engaged research (CEnR) is increasingly recognized as a critical component of effective clinical and translational science, particularly for improving the relevance, equity, and impact of research conducted at academic medical centers [1–3]. By fostering bi-directional partnerships between academic institutions and the communities that they serve, CEnR supports translational goals such as improved uptake of evidence, responsiveness to community priorities, and reductions in health disparities, grounded in the principles of community-based participatory research [4, 5]. Despite this recognition, many academic health centers continue to face challenges in systematically supporting and sustaining CEnR.

Prior studies have documented persistent institutional barriers including misaligned incentive structures, limited administrative flexibility, inadequate financial mechanisms to support community partners, and variability in leadership support [2, 6, 7]. These challenges reflect the fact that CEnR represents a shift from traditional, investigator-driven research models towards partnership-based approaches that requires different organizational competencies, policies, and workflows. As a result, existing institutional infrastructures may be insufficiently equipped to support CEnR.

Recent scholarship has advanced the science of community engagement by centering on community partner perspectives [8], emphasizing process of co-creation [9], shared power [10], reflexivity [11], and collective empowerment [12,13] as central to community-academic partnerships producing meaningful research outcomes. This literature, situated largely at the partnership or the project level, has been instrumental in articulating how engagement processes contribute to trust and relevance in CEnR. At the same time, these partnership processes do not occur in isolation; they are influenced by institutional environment that can either enable or constrain their enactment [14]. There is also a need to examine the roles of community outreach and engagement offices/centers, supported in recent years through Clinical and Translational Science Centers [1] and Cancer Centers [15] with goals to engage communities served by the academic medical centers, disseminate research activities, and conduct outreach and education for the community. Building on the strong foundation in community engagement science, we highlight the critical need for an institutional understanding of the assets and opportunities to support CEnR. Much of the existing literature, however, has examined these barriers from a single interest holder perspective or has relied on qualitative or quantitative methods alone which limits the ability to fully characterize institutional context and identify actionable opportunities for improvements meaningful to both academic and community partners.

Supported by the Patient Centered Outcomes Research Institute (PCORI) Engagement Award, we previously tested the feasibility of a data-driven, partnership-engaged, Engage for Equity PLUS (E^2^PLUS) intervention to understand and enhance institutional internal capacity to support CEnR, strengthen internal and external structures and processes to enhance institutional commitment to health equity through joint patient and community decision-making, and shared governance in research [16].

In this paper, we present a mixed-methods approach to integrate measurable institutional conditions with partner experiences. Using a sequential exploratory mixed-methods design, this study examined institutional support for CEnR across three geographic sites of an academic medical center. The qualitative research objective was to explore how academic investigators, institutional staff, and community partners perceive and experience institutional structures, policies, processes, and relational dynamics that influence CEnR. The quantitative objective was to assess across a broader sample of academic and community partners the perceptions of institutional commitment, trustworthiness, leadership support, policies, and infrastructure for CENR, informed by domains identified in the qualitative phase. The mixed-methods objective was to identify areas of convergence and divergence between academic and community perspectives, deepen understanding of site-specific institutional context, and inform actionable strategies to strengthen institutional support for CEnR within academic medical centers.

## Materials and methods

### Study design

This was a sequential exploratory mixed-methods study design for a pragmatic inquiry into the context of the academic medical center in order to identify strategies that can support institutional learning and translation impact [17]. In the first phase, we collected qualitative data through interviews with institutional academic leaders, community-engaged researchers and staff from community-facing offices, and focus groups with community members. In the second phase, we used the qualitative data to inform the adaptation of a quantitative data collection instrument [18], using an online REDCap survey of academic and community members. Qualitative findings were used to refine survey domains, ensure contextual relevance, and incorporate community and academic partner-identified priorities related to the institutional and operational support for CEnR. Quantitative survey data were subsequently used to examine the distribution of perceptions identified qualitatively and to assess differences between academic and community respondents. Integration occurred at multiple points: during survey development, through comparison of qualitative themes and quantitative patterns, and interpretation of findings to generate institutional-level recommendations.

### Theoretical framework

The institutional assessment was informed by an aggregation of constructs from two key frameworks: the CBPR model [19] and the Assessing Community Engagement Model by the National Academies of Science, Engineering, and Medicine [20]. In addition, the assessment was guided by an in-depth understanding of contextual assessments common in the implementation science literature and often included in its theories, models and frameworks [21]. Detailed information about the underlying theoretical framework is presented elsewhere [22]. Together, these frameworks informed the development of the qualitative data collection instruments with specified domains outlined in Table 1. The qualitative findings then supported the adaptation of a published institutional survey [18] that was refined for this project.

**Table 1.** Domains of inquiry for the qualitative and quantitative data

Institutional Leadership Interviews	Community, researcher, and staff focus group discussions	Institutional Multi-Sector Survey (all participants)
Organizational structure and decision making at the institutional level Leadership style as it related to organizational governance Aspirational focus for supporting research Perceptions about institutional status, power and resources Key initiatives supporting institutional transformation and innovation Diversity, equity, and inclusion initiative, and anti-racism initiatives Value-add for community-engaged, CBPR Integration with institutional offices of community outreach and engagement Governance at the level of the clinical translational science center	**Community members** Involvement in CEnR Perceptions about institutional support for CEnR Key barriers to operationalizing equity-oriented CEnR Strategies to strengthen CEnR Aspirational focus for supporting research Key initiatives supporting institutional transformation and innovation **Researchers** Status and reputation of the institution Institutional centers supporting CEnR Key barriers to operationalizing equity-oriented CEnR Strategies to strengthen CEnR Key initiatives supporting institutional transformation and innovation Engagement of community in institutional decision making and research	Involvement in CEnR Perceptions about institutional support mission, vision, and values Institutional trustworthiness (communications and coordination, planning and action, value-add for community engagement, and sustainable engagement) Institutional reputation, culture, and climate for CEnR Leadership support for CEnR Policies and practices (fiscal and administrative support, community training and support, and evaluation and standards) Involvement as a community advisory board Perceptions of race and racism

### Participant recruitment and data collection

The research team from University of New Mexico (UNM) led the design and development of the data collection instruments with leaders from the institutional Clinical and Translational Science Award center, which operates in three geographically distinct sites in the United States. Together the UNM team and the CTSA leaders formed a core champion team that also included community members, researchers, and staff. The champion team collectively served in an advisory role to the data collection protocols as well as throughout the data interpretation phase. All data collection instruments (i.e., interview guides, focus group discussion guides, and survey) were adapted from prior work on barriers and facilitators embedded in institutional contexts to be more congruent with this academic medical center and the evaluation goals [18,22]. In addition, we added 20 items that were generated from the Principles of Trustworthiness put forth by the American Association of Medical Colleges, Center for Health Justice.[23] While data collection was led by the UNM team, the champion team led the participant recruitment based on existing relationships with senior leadership, community-engaged researchers, staff from community-facing offices, and community partners from institutional advisory boards and from CEnR partnerships. Study protocol and all data collection instruments were approved by the institutional review boards of the University of New Mexico Health Sciences Center and the academic medical center.

Data were collected from August through October 2023. Interviews and focus groups were conducted both in-person and on video-conferencing platforms. We conducted in-depth interviews with academic leaders, researchers, and staff (*n* = 54) and focus groups’ discussions (*N* = 4) with community members (*n* = 33). Using rapid analytic approaches, the themes from these qualitative data informed the adaptation of the institutional survey which was administered through REDCap [24]. Survey invitations were sent to 175 leaders, investigators, and community partners affiliated (i.e., those serving on institutional advisory boards or engaged in a CEnR partnership) with the academic medical center. A total of five rounds of invitations were sent between September 20, 2023, and October 6, 2023. We received 85 responses, for a response rate of 49%. Community participants received an electronic gift card for participation whereas academic participants were not remunerated.

### Data analyses

We conducted rapid qualitative analysis [25] on the qualitative data from 87 people affiliated with the academic medical center that were collected through leadership interviews, researcher, staff, and community focus groups. Following interviews and focus groups at each of the three sites, trained members of the research team (PA, AK, NW) generated analytic memos that synthesized key points related to theory-informed domains, documented emergent insights, and captured illustrative quotations. To enhance analytic rigor, participatory sense-making discussions were held with additional members of the academic health center team (IS, MLW) to compare interpretations and resolve differences through consensus. Findings from the qualitative phase were used to adapt and refine the institutional survey instrument. All individuals consenting to the survey were included in the analysis. Descriptive statistics were calculated for all survey items, including frequencies for Likert-type responses, stratified by community versus academic respondents. Group differences in response distributions were examined using Mann–Whitney *U* tests, excluding missing and “don’t know” responses. To facilitate interpretability, we present z-statistics for significance tests. A negative *z*-statistic indicates that the ranked sum for community members was higher than that of institutional members. Positive *z*-scores, on the other hand, indicate higher ranked sums among institutional participants. Depending on the response options for each question, higher values for each item is either associated with a higher level of agreement (e.g., “Strongly Agree”) or frequency (e.g., “Always”). Following survey data analyses, qualitative data were revisited to examine how interview and focus group findings explained or contextualized. This interpretive return to the data supported the mixed methods integration during the interpretation phase.

## Results

### Findings from the interviews and focus groups

#### Theme 1: Establishing a linkage between clinical care and research

“[The institution] does have a good reputation, and most people feel like physicians know what they are doing” which people said helps with cross-institutional collaborations and trust in clinical care, though many community members suggested the need for the institution to still invest more in relationships with organizations so that uninsured patients can receive high-quality care, which would also impact the support for research.

Data from researchers and staff indicate that the institution doesn’t yet see CEnR as a part of the “continuum of care” for what is delivered in the clinic. According to community members, it is important to invest not just in research but, “*in its people so that people can see that [the institution] will honor the community and not just go get blood [for the research*]” – (Community member) Staff relayed anecdotes of conversations with individuals recruited into research studies who perceive that they did not have access to the clinical practice: “*I’m good enough for research, but not good enough for care*” (Staff member) These clinical barriers have made recruiting for research more difficult.

In leadership interviews, several leaders mentioned that the institution differentiates itself from other academic health centers in that, “*we really view research and discovery as way to improve patient care.*” Many interviewees brought up the fact that it is a physician-led organization, operating like “*a tricycle with clinical care being the big front wheel, supported by research and education*.” To integrate into the Clinic, there is a need to examine the intersection of CEnR with the concept of a “*learning healthcare system*” where community engagement and health equity research can play an important role in addressing the main focus of unmet patient needs as described by a leader below,

*We also talk about the needs of the patient come first. That’s our primary values. That’s what roots us. When we think about research, we think about unmet patient needs*. (Institutional Leader)

#### Theme 2: Leveraging the institutional reputation in the community

Many community members stated that the institution has to balance the needs of patients seeking care from around the world and the needs of communities in the institution’s back yard: “*There is still a lot of mistrust in healthcare*.” (community member). “*There’s two perspectives, there is the [institutional] perspective and the community health perspective, and they are too far apart*” said a community member, encouraging the institution’s approach to the community to be less transactional. They also encourage the clinic to invest in continuous engagement with community organizations that have been working hard to build trust in the community.

Many researchers and leaders in the institution agreed with the community members perceptions that, “*The main thrust of [the institution] is serious and complex disease*” and “*Our DNA is still in serious and complex care.*” Several staff members mentioned that there is a lot of “*brand name*” trust that the [institution] generates in the community, “*If you google anything about medicine, [the institution] pops up first*.” Some researchers indicated that this focus on complexity sometimes makes it difficult to collaborate with local communities on research and practice priorities. One leader said, “*If [the institution] leads, others will follow*” while another mentioned “*If anyone can figure out how to make sure people have access to care, it’s [the institution]*” – (Institutional Leader).

#### Theme 3: Going beyond community engagement as a grant mandate

Many community members questioned, “*How much is the institution genuinely interested in community participation and how much of it is done simply to check a box?”* Several thought that the community engagement activities were happening because it is required of them through federal grants. Community members perceived that the institution is not always prepared to support CEnR and were not sure that the work is being “*translated to a larger institutional policy and really being institutionalized in ways that can be felt outside research*.” Authentic and meaningful approach to CEnR would mean that the institution invests in building community capacity through research outcomes and provide sustainable solutions through CEnR. They also encouraged, genuine and authentic interactions including “*breaking bread with the community*,” showing up in-person and “*looking people in the eye”* which was hard to do online, although many community members also supported hybrid approaches. Community members shared that researchers who have a passion for a certain field are important, but other times it would be great for them to look at ways in which they could address community priorities; often it seemed that “*[the institution] is not asking what new research should be done.*” (Community member).

For authentic engagement, some researchers suggested that the institution should create space for the community to define their needs and assets and generate a sustained presence in the community after the research is over through newsletters and communications. Leadership agreed that community engagement was a key priority for federal research which meant that these activities were happening across the institution, but they also acknowledged that “*it was typical of the institution to have all these activities siloed and not coordinated in a larger effort… “where is the front door?”* Some leaders acknowledged that CEnR was a “field in itself” and they had to learn a lot; most investigators “don’t really understand the field, and so they bring on someone else, and they don’t understand it either” acknowledging that there was a lot to learn about CEnR. One leader acknowledged the need to move beyond the grants, as stated below:

*We like grants…but it is instantly wrong for me to say, ‘well, this is what [federal grants] require, therefore this is what we should do’ and really that’s not what we should be doing at all… We should align when we can but we should be driven by what [the institution] …is well-suited to do. If that aligns with [federal grants], fine. If it doesn’t, we are going to have to figure out how to do both and keep our grants.* (Institutional Leader)

#### Theme 4: Addressing complexity around health equity in CEnR

Addressing the complexity around promoting health equity and prioritizing community is “*more than any one health system can do*” given the mistrust and fear in the community which would require a “*360-degree approach*” according to community member participants. Addressing community priorities requires an awareness of “*issues of trust and communication and transparency*” which is lacking when community engagement is conducted “*in a very superficial manner for research*” Community members mentioned that CEnR investigators have “*to meet the community where the community is*” and be transparent with what you want from the community; they also need to develop communication skills and “*a humility that needs to be embedded in the process*” of community engagement, further elucidating the complexity.

Several researchers pointed out that conducting CEnR requires a lot more intentionality and effort than other research approaches. There is a need to address historical tensions in the community, rebuilding trust, and yet this doesn’t support their promotion and tenure, as noted by an investigator below,

*So the entire structure down to even how we are rated in performance of our job does not align with community-based research. It’s a heavy emphasis on timelines for basic scientists to get things out, which is typically faster than it takes people like us who have to build these relationships in the community. It becomes even more challenging when there have been some historic circumstances that have caused tension with us in the community, and we have to now go back in and try to rebuild that structure.* [Investigator]

Per one investigator, “*there is nothing more complex…than social determinants of health in the context of chronic disease*,” while mentioning the opportunity for the institution to leverage its reputation in the community and expertise towards community health. Leadership perceived the complexity around health equity research in relation to social determinants of health by noting the need to address the research to practice gap, which would require coordination with other community-based organizations. One leader noted the need to encourage scientists to undertake research that “*allows you to change paradigms vs research that is there to confirm what most of us already knew, 20 years ago.*”

#### Theme 5: Institutionalized prioritization and support for CEnR

Community members noted that there are many investigators driven by genuine interest in the community, but the “*institution as a whole is driven by other factors*.” Many community members noted themselves to be, “*trusted messengers for the community*” which is a reputation that has taken them years to build in the community and they needed to see institutional investment in CEnR outside of waiting for NIH funding. “*You can’t do lip service to community engagement*;” community members said it needed to be *“central, until every department must look at itself and say,* “*How are we involved in the community? What are we doing to improve health outcomes of the minority populations in the community*?” They encouraged the institution to *“take risks and invest where it matters*” for more impactful change and sustainable community solutions that showcase their intentions. Although, many noted that changing for organizations can be hard, *“we as humans, don’t do it naturally, so how would a big organization do it naturally?”*

Researchers mentioned primarily relying on their own grant success to convince leadership of the value of CEnR. They suggested that institutionalization of CEnR is possible by giving voice and infrastructure support to the investigators leading this work, including sustained fiscal support for ongoing partnerships between funding cycles and addressing internal structural barriers (IRB approvals, mechanics of compensation for community partners, etc.) to reduce impact on fragile relationships in the community for researchers.

Leaders described challenges in aggregating a common vision for CEnR across the three national sites. They mentioned the need for interagency coordination and communications to strengthen community engagement and, most importantly, to scale it across sites. Some mentioned that not all leaders understood community engagement and that “*community engagement kind of needs to be one voice*.” Many described the importance of structures such as the advisory boards, Community Engagement Studios, and the central offices for Community Engagement at the Cancer Center and the Clinical Translations Science Center. However, they also cautioned against over-reliance on these structures, mentioning the need for infrastructure investment to bolster the workforce with individuals that understand what it takes to engage, recruit, and retain participants since community engagement and CEnR is “*joined at the hip,*” according to a researcher.

### Findings from the institutional survey

Of the 85 people who responded to the survey, 60% identified as members of communities that interacted with the institution, and 40% were members of the institution. Nearly half of respondents (43.1%) identified themselves as being involved in CBPR and having 1–5 years of experience in CEnR (46.5%). Detailed sample demographic information is presented in Table 2. Tables 3, 4, 5, and 6 provide descriptive statistics for the survey, including the results of Mann Whitney U tests to assess significant differences in response distributions between community and institutional members. Select findings that expand on the qualitative findings are summarized here.

**Table 2.** Demographic characteristics of the multi-sector survey respondents

	Overall (*n* = 85)		Community (*n* = 51)		Academic (*n* = 34)	
Characteristics	*N*	%	*N*	%	*N*	%
Sites						
Southwest	18	21.2	10	19.6	8	23.5
Southeast	22	25.9	15	29.4	7	20.6
Midwest	40	47.1	23	45.1	17	50.0
Midwest community health system	3	3.5	2	3.9	1	2.9
Missing	2	2.4	1	2.0	1	2.9
Race						
American Indian or Alaska Native	2	2.4	1	2.0	1	2.9
Black or African American	31	36.5	20	39.2	11	32.4
White	46	54.1	28	54.9	18	52.9
Prefer not to say	4	4.7	2	3.9	2	5.9
Other	1	1.2	0	0	1	2.9
Missing	1	1.2	0	0	1	2.9
Ethnicity						
Hispanic or Latino	14	16.5	11	21.6	3	8.8
Non-Hispanic or Latino	67	78.8	37	72.5	30	88.2
Prefer not to say	2	2.4	1	2.0	1	2.9
Missing	2	2.4	2	3.9	0	0
Gender						
Men	29	34.1	18	35.3	11	32.4
Women	53	62.4	31	60.8	22	64.7
Prefer not to say	2	2.4	1	2.0	1	2.9
Prefer to self-describe	1	1.2	1	2.0	0	0
Missing	0	0	0	0	0	0
Academic role						
Career scientist	–	–	–	–	1	2.9
Collaborative scientist	–	–	–	–	1	2.9
Clinician engaged in research	–	–	–	–	3	8.8
Clinician investigator	–	–	–	–	2	5.9
Clinician	–	–	–	–	2	5.9
Other	–	–	–	–	14	41.2
Missing	–	–	–	–	11	32.4
Engagement across CEnR continuum						
Community-informed	6	10.3	6	17.1	0	0
Community consultation	8	13.7	7	20.0	1	4.3
Community participation	17	29.3	9	25.7	8	34.8
Community-initiated	1	1.7	0	0	1	4.3
Community-based participatory	25	43.1	13	37.1	12	52.2
Community transfer of power	0	0	0	0	0	0
I am not sure	0	0	0	0	0	0
Other	1	1.7	0	0	1	4.3
Length of CEnR work						
Less than 1 year	8	13.7	6	17.1	2	8.7
1–5 years	27	46.5	16	45.7	11	47.8
6–10 years	11	18.9	8	22.9	3	13.0
11–15 years	9	15.5	5	14.3	4	17.4
16+ years	3	5.1	0	0	3	13.0

**Table 3.** Mission, vision, and values for the institution

	Overall (*n* = 85)		Community (*n* = 51)		Academic (*n* = 34)		
Statements	*n*	%	*n*	%	*n*	%	*z*-Statistic
1. Do you believe [the institution] demonstrates a commitment to health equity?							
Never	0	0	0	0	0	0	−3.045 (*p* = 0.002)
Almost never	4	4.7	2	3.9	2	5.9	
Sometimes	33	38.8	13	25.5	20	58.8	
Almost always	24	28.2	17	33.3	7	20.6	
Always	17	20.0	14	27.5	3	8.8	
Don’t know	2	2.4	2	3.9	0	0	–
Missing	5	5.9	3	5.9	2	5.9	–
2. Do you believe [the institution] takes action to demonstrate its commitment to health equity?							
Never	0	0	0	0	0	0	−3.443 (*p* < 0.001)
Almost never	8	9.4	2	3.9	6	17.6	
Sometimes	35	41.2	16	31.4	19	55.9	
Almost always	19	22.4	15	29.4	4	11.8	
Always	16	18.8	13	25.5	3	8.8	
Don’t know	2	2.4	2	3.9	0	0	–
Missing	5	5.9	3	5.9	2	5.9	–
3. Do you believe [the institution] demonstrates a commitment to conducting CEnR							
Never	0	0	0	0	0	0	−3.903 (*p* < 0.001)
Almost never	9	10.6	4	7.8	5	14.7	
Sometimes	27	31.8	9	17.6	18	52.9	
Almost always	21	24.7	14	27.5	7	20.6	
Always	22	39.2	20	39.2	2	5.9	
Don’t know	1	2.0	1	2.0	0	0	–
Missing	5	5.9	3	5.9	2	5.9	–
4. Do you believe [the institution] takes action to demonstrate its commitment to CEnR?							
Never	0	0	0	0	0	0	−3.729 (*p* < 0.001)
Almost never	9	10.6	3	5.9	6	17.6	
Sometimes	31	36.5	13	25.5	18	52.9	
Almost always	18	21.2	12	23.5	6	17.6	
Always	20	23.5	18	35.3	2	5.9	
Don’t know	2	2.4	2	3.9	0	0	–
Missing	5	5.9	3	5.9	2	5.9	–

**Table 4.** Institutional trustworthiness

Domains/statements	Overall		Community		Academic		
Domain 1: Communications and coordination	*N*	%	*N*	%	*N*	%	*z*-statistic
**1. [Institutional] researchers communicate organization-wide policies and expectations when working with the community**							
Strongly disagree	1	1.2	1	2.0	0	0	−1.909 (*p* = 0.056)
Disagree	11	12.9	4	7.8	7	20.6	
Neither agree nor disagree	15	17.6	7	13.7	8	23.5	
Agree	27	31.8	19	37.3	8	23.5	
Strongly agree	17	20.0	12	23.5	5	14.7	
Don’t know	7	8.2	3	5.9	4	11.8	–
Missing	7	8.2	5	9.8	2	5.9	–
**2. [The institution] demonstrates an understanding across the organization that community engagement in research is valuable**							
Strongly disagree	5	5.9	3	5.9	2	5.9	−3.203 (*p* = 0.001)
Disagree	19	22.4	5	9.8	14	41.2	
Neither agree nor disagree	13	15.3	5	9.8	8	23.5	
Agree	19	22.4	17	33.3	2	5.9	
Strongly agree	19	22.4	14	27.5	5	14.7	
Don’t know	3	3.5	2	3.9	1	2.9	–
Missing	7	8.2	5	9.8	2	5.9	–
**3. [The institution] demonstrates a commitment to adequately compensate community partners for their work**							
Strongly disagree	2	2.0	1	2.4	1	2.9	−3.278 (*p* = 0.001)
Disagree	14	9.8	5	16.5	9	26.5	
Neither agree nor disagree	17	13.7	7	20.0	10	29.4	
Agree	25	27.5	14	29.4	11	32.4	
Strongly agree	16	29.4	15	18.8	1	2.9	
Don’t know	5	9.8	5	5.9	0	0	–
Missing	6	7.8	4	7.1	2	5.9	–
**4. [Institutional] researchers support honest communication with the community**							
Strongly disagree	2	2.4	2	3.9	0	0	−0.794 (*p* = 0.427)
Disagree	3	3.5	2	3.9	1	2.9	
Neither agree nor disagree	15	17.6	7	13.7	8	23.5	
Agree	36	42.4	20	39.2	16	47.1	
Strongly agree	20	23.5	14	27.5	6	17.6	
Don’t know	3	3.5	2	3.9	1	2.9	–
Missing	6	7.1	4	7.8	2	5.9	–
**5. [The institution] routinely engages community organizations and community leaders to assess potential new partnerships**							
Strongly disagree	1	1.2	1	2.0	0	0	−0.398 (*p* = 0.691)
Disagree	8	9.4	7	13.7	1	2.9	
Neither agree nor disagree	18	21.2	7	13.7	11	32.4	
Agree	34	40.0	19	37.3	15	44.1	
Strongly agree	17	20.0	12	23.5	5	14.7	
Don’t know	1	1.2	1	2.0	0	0	–
Missing	6	7.1	4	7.8	2	5.9	–
**6. [Institutional] researchers demonstrate clarity and intentionality about working with the community**							
Strongly disagree	3	3.5	1	2.0	2	5.9	−1.910 (*p* = 0.056)
Disagree	10	11.8	6	11.8	4	11.8	
Neither agree nor disagree	17	20.0	8	15.7	9	26.5	
Agree	26	30.6	13	25.5	13	38.2	
Strongly agree	21	24.7	17	33.3	4	11.8	
Don’t know	2	2.4	2	3.9	0	0	–
Missing	6	7.1	4	7.8	2	5.9	–
**7. [Institutional] researchers co-develop expectations and goals with the community**							
Strongly disagree	3	3.5	2	3.9	1	2.9	−2.171 (*p* = 0.030)
Disagree	11	12.9	5	9.8	6	17.6	
Neither agree nor disagree	22	25.9	11	21.6	11	32.4	
Agree	20	23.5	11	21.6	9	26.5	
Strongly agree	14	16.5	13	25.5	1	2.9	
Don’t know	9	10.6	5	9.8	4	11.8	–
Missing	6	7.1	4	7.8	2	5.9	–
**8. [Institutional] researchers create an open, honest, and accessible environment where community members feel safe and are able to provide feedback**							
Strongly disagree	1	1.2	1	2.0	0	0	−1.392 (*p* = 0.164)
Disagree	9	10.6	8	15.7	1	2.9	
Neither agree nor disagree	18	21.2	5	9.8	13	38.2	
Agree	31	36.5	16	31.4	15	44.1	
Strongly agree	16	18.8	14	27.5	2	5.9	
Don’t know	3	3.5	2	3.9	1	2.9	–
Missing	7	8.2	5	9.8	2	5.9	–
Domain 2: Planning and action							
**9. [Institutional] researchers have taken the time to understand local knowledge and history of the communities with whom they work**							
Strongly disagree	4	4.7	2	3.9	2	5.9	−1.489 (*p* = 0.136)
Disagree	15	17.6	9	17.6	6	17.6	
Neither agree nor disagree	20	23.5	10	19.6	10	29.4	
Agree	22	25.9	12	23.5	10	29.4	
Strongly agree	12	14.1	11	21.6	1	2.9	
Don’t know	3	3.5	2	3.9	1	2.9	–
Missing	9	10.6	5	9.8	4	11.8	–
**10. There are concrete ways in which [institutional] researchers have incorporated community expertise in their work**							
Strongly disagree	2	2.4	1	2.0	1	2.9	−0.591 (*p* = 0.555)
Disagree	5	5.9	4	7.8	1	2.9	
Neither agree nor disagree	15	17.6	9	17.6	6	17.6	
Agree	38	44.7	19	37.3	19	55.9	
Strongly agree	15	17.6	12	23.5	3	8.8	
Don’t know	1	1.2	1	2.0	0	0	–
Missing	9	10.6	5	9.8	4	11.7	–
**11. [The institution’s] strategy reflects how CEnR aligns with the assets, needs, and goals of the community.**							
Strongly disagree	4	4.7	2	5.9	2	3.9	−2.088 (*p* = 0.037)
Disagree	15	17.6	8	23.5	7	13.7	
Neither agree nor disagree	21	24.7	10	29.4	11	21.6	
Agree	21	24.7	7	20.6	14	27.5	
Strongly agree	12	14.1	2	5.9	10	19.6	
Don’t know	3	3.5	1	2.9	2	3.9	–
Missing	9	10.6	4	11.8	5	9.8	–
**12. [Institutional] researchers follow up on their words with meaningful action**							
Strongly disagree	3	3.5	0	0	3	5.9	−0.516 (*p* = 0.606)
Disagree	11	12.9	4	11.8	7	13.7	
Neither agree nor disagree	16	18.8	8	23.5	8	15.7	
Agree	27	31.8	14	41.2	13	25.5	
Strongly agree	16	18.8	3	8.8	13	25.5	
Don’t know	3	3.5	1	2.9	2	3.9	–
Missing	9	10.6	4	11.8	5	9.8	–
**13. [Institutional] plan for action often holds the organizational leadership accountable**							
Strongly disagree	6	7.1	4	11.8	2	3.9	−2.570 (*p* = 0.010)
Disagree	12	14.1	6	17.6	6	11.7	
Neither agree nor disagree	23	27.1	12	35.2	11	21.6	
Agree	18	21.2	4	11.8	14	27.5	
Strongly agree	7	8.2	1	2.9	6	11.8	
Don’t know	10	11.8	3	8.8	7	13.7	–
Missing	9	10.6	4	11.8	5	9.8	–
**14. [The institution’s] plan for action is co-developed with the community**							
Strongly disagree	4	4.7	2	3.9	2	5.9	−2.000 (*p* = 0.046)
Disagree	15	17.6	9	17.6	6	17.6	
Neither agree nor disagree	21	24.7	9	17.6	12	35.3	
Agree	19	22.4	12	23.5	7	20.6	
Strongly agree	10	11.8	10	19.6	0	0	
Don’t know	7	8.2	4	7.8	3	8.8	–
Missing	9	10.6	5	9.8	4	11.8	–
**Domain 3: value-added of CEnR**							
**15. [Institutional] researchers consider that diversity is more than race and ethnicity**							
Strongly disagree	2	2.4	2	3.9	0	0	−1.425 (*p* = 0.154)
Disagree	3	3.5	1	2.0	2	5.9	
Neither agree nor disagree	14	16.5	6	11.8	8	23.5	
Agree	31	36.5	18	35.3	13	38.2	
Strongly agree	21	24.7	15	29.4	6	17.6	
Don’t know	4	4.7	3	5.9	1	2.9	–
Missing	10	11.8	6	11.8	4	11.8	–
**16. [Institutional] researchers understand and value the multiple identities and lived experiences of community members**							
Strongly disagree	2	2.4	2	3.9	0	0	−0.699 (*p* = 0.485)
Disagree	9	10.6	4	7.8	5	14.7	
Neither agree nor disagree	9	10.6	6	11.8	3	8.8	
Agree	33	38.8	17	33.3	16	47.1	
Strongly agree	18	21.2	13	25.5	5	14.7	
Don’t know	5	5.9	4	7.8	1	2.9	–
Missing	9	10.6	5	9.8	4	11.8	–
**17. [Institutional] researchers often meet and interact with community members where they work and live.**							
Strongly disagree	4	4.7	2	3.9	2	5.9	−0.699 (*p* = 0.485)
Disagree	9	10.6	5	9.8	4	11.8	
Neither agree nor disagree	17	20.0	8	15.7	9	26.5	
Agree	28	32.9	17	33.3	11	32.4	
Strongly agree	12	14.1	10	19.6	2	5.9	
Don’t know	6	7.1	4	7.8	2	5.9	–
Missing	9	10.6	5	9.8	4	11.8	–
**Domain 4: Sustainable Engagement**							
**18. [Institutional] researchers continue to build and maintain trust within the community**							
Strongly disagree	2	2.4	2	3.9	0	0	−0.959 (*p* = 0.337)
Disagree	6	7.1	3	5.9	3	8.8	
Neither agree nor disagree	11	12.9	7	13.7	4	11.8	
Agree	38	44.7	19	37.2	19	55.9	
Strongly agree	14	16.5	12	23.5	2	5.9	
Don’t know	4	4.7	2	3.9	2	5.9	–
Missing	10	11.8	6	11.8	4	11.8	–
**19. Evaluation and dissemination plans are built into partnerships with community members**							
Strongly disagree	3	3.5	2	3.9	1	2.9	−1.525 (*p* = 0.127)
Disagree	8	9.4	5	9.8	3	8.8	
Neither agree nor disagree	16	18.8	7	13.7	9	26.5	
Agree	25	29.4	14	27.5	11	32.4	
Strongly agree	14	16.5	12	23.5	2	5.9	
Don’t know	8	9.4	5	9.8	3	8.8	–
Missing	11	12.9	6	11.8	5	14.7	–
**20. [Institutional] researchers find ways to engage community partners when not tied to a specific project**							
Strongly disagree	7	8.2	3	5.9	4	11.8	2.020 (*p* = 0.043)
Disagree	9	10.6	5	9.8	4	11.8	
Neither agree nor disagree	19	22.4	9	17.6	10	29.4	
Agree	22	25.9	11	21.6	11	32.4	
Strongly agree	13	15.3	12	23.5	1	2.9	
Don’t know	4	4.7	4	7.8	0	0	–
Missing	11	12.9	7	13.7	4	11.8	–

**Table 5.** Institutional reputation, culture, and climate for community-engaged research

	Overall		Community		Academic		
Domains/Statements	*N*	%	*N*	%	*N*	%	*z*-statistic
**1. [The institution] is recognized for its reputation in CEnR**							
Strongly disagree	8	9.4	2	3.9	6	17.6	−4.122 (*p* < 0.001)
Disagree	12	14.1	4	7.8	8	23.5	
Neither agree nor disagree	19	22.4	10	19.6	9	26.5	
Agree	16	18.8	15	29.4	1	2.9	
Strongly agree	9	10.6	8	15.7	1	2.9	
Don’t know	6	7.1	4	7.8	2	5.9	–
Missing	15	17.6	8	15.7	7	20.6	–
**2. [The institution] invites community input in strategic planning at the leadership level**							
Strongly disagree	6	7.1	1	2.0	5	14.7	−2.501 (*p* = 0.012)
Disagree	13	15.3	6	11.8	7	20.6	
Neither agree nor disagree	17	20.0	10	19.6	7	20.6	
Agree	16	18.8	11	21.5	5	14.7	
Strongly agree	6	7.1	5	9.8	1	2.9	
Don’t know	11	12.9	9	17.6	2	5.9	–
Missing	16	18.8	9	17.6	7	20.6	–
**3. [Institutional] researchers are evaluated and promoted based on criteria that values CEnR**							
Strongly disagree	11	12.9	3	5.9	8	23.5	−3.876 (*p* < 0.001)
Disagree	6	7.1	1	2.0	5	14.7	
Neither agree nor disagree	16	18.8	10	19.6	6	17.6	
Agree	14	16.5	12	23.5	2	5.9	
Strongly agree	5	5.9	5	9.8	0	0	
Don’t know	19	22.4	12	23.5	7	20.6	–
Missing	14	16.5	8	15.7	6	17.6	–
**4. [Institutional] researchers are willing to change how they conduct research in response to community feedback**							
Strongly disagree	3	3.5	3	5.9	0	0	−1.286 (*p* = 0.198)
Disagree	4	4.7	1	2.0	3	8.8	
Neither agree nor disagree	10	11.8	5	9.8	5	14.7	
Agree	35	41.2	18	35.2	17	50.0	
Strongly agree	12	14.1	10	19.6	2	5.9	
Don’t know	6	7.1	5	9.8	1	2.9	–
Missing	15	17.6	9	17.6	6	17.6	–
**5. [Institutional] researchers and community partners regularly reflect on their work together**							
Strongly disagree	2	2.4	2	3.9	0	0	−2.053 (*p* = 0.040)
Disagree	12	14.1	4	7.8	8	23.5	
Neither agree nor disagree	15	17.6	8	15.7	7	20.6	
Agree	23	27.1	13	25.5	10	29.4	
Strongly agree	11	12.9	10	19.6	1	2.9	
Don’t know	8	9.4	6	11.8	2	5.9	–
Missing	14	16.5	8	15.7	6	17.6	–
**6. Research staff frustration is common at [the institution]**							
Strongly disagree	1	1.2	1	2.0	0	0	2.259 (*p* = 0.024)
Disagree	4	4.7	3	5.9	1	2.9	
Neither agree nor disagree	20	23.5	12	23.5	8	23.5	
Agree	12	14.1	4	7.8	8	23.5	
Strongly agree	10	11.8	3	5.9	7	20.6	
Don’t know	24	28.2	20	39.2	4	11.8	–
Missing	14	16.5	8	15.7	6	17.6	–
**7. [The institution] actively mobilizes community partnerships to address environmental, societal, and economic conditions that influence health**							
Strongly disagree	3	3.5	2	3.9	1	2.9	−1.401 (*p* = 0.161)
Disagree	9	10.6	5	9.8	4	11.8	
Neither agree nor disagree	16	18.8	6	11.8	10	29.4	
Agree	27	31.8	17	33.3	10	29.4	
Strongly agree	9	10.6	7	13.7	2	5.9	
Don’t know	6	7.1	5	9.8	1	2.9	–
Missing	15	17.6	9	17.6	6	17.6	–
**8. [The institution] develops interventions with community partners to address health inequities**							
Strongly disagree	2	2.4	1	2.0	1	2.9	−1.023 (*p* = 0.306)
Disagree	11	12.9	6	11.8	5	14.7	
Neither agree nor disagree	11	12.9	6	11.8	5	14.7	
Agree	33	38.8	18	35.3	15	44.1	
Strongly agree	10	11.8	8	15.7	2	5.9	
Don’t know	4	4.7	4	7.8	0	0	–
Missing	14	16.5	8	15.7	6	17.6	–
**9. [The institution] contributes to innovative solutions to address health inequities**							
Strongly disagree	3	3.5	1	2.0	2	5.9	−1.282 (*p* = 0.200)
Disagree	6	7.1	4	7.8	2	5.9	
Neither agree nor disagree	20	23.5	10	19.6	10	29.4	
Agree	26	30.6	17	33.3	9	26.5	
Strongly agree	11	12.9	8	15.7	3	8.8	
Strongly disagree	4	4.7	3	5.9	1	2.9	–
Missing	15	17.6	8	15.7	7	20.6	–
**10. [Institutional] researchers have the necessary training and resources to conduct CEnR**							
Strongly disagree	2	2.4	1	2.0	1	2.9	−2.316 (*p* = 0.021)
Disagree	14	16.5	5	9.8	9	26.5	
Neither agree nor disagree	13	15.3	7	13.7	6	17.6	
Agree	20	23.5	12	23.5	8	23.5	
Strongly agree	15	17.6	12	23.5	3	8.8	
Strongly disagree	7	8.2	6	11.8	1	2.9	–
Missing	14	16.5	8	15.7	6	17.6	–

When asked about the institution’s commitment to health equity and CEnR, most respondents acknowledged the institution’s stated commitment for both health equity and CEnR. The discrepancy apparent between participants’ perception of the institution’s stated commitment to health equity (48.2% rated always or almost always; Table 3, Q1) and their action to demonstrate this commitment (41.2% rated always or almost always; Table 3, Q2) underscores the theme that emerged from the qualitative data regarding the complexity associated with addressing social determinants of health and promoting health equity. While community members were more likely than institutional respondents to perceive that these commitments were demonstrated in practice, institutional respondents may be more attuned to the challenges of translating aspirational values into actionable institutional strategies.

Institutional trustworthiness was assessed across four subdomains: communication practices, planning and action strategies, value assigned to community perspectives, and efforts to promote sustainable engagement. Items separately assessed perceptions of the trustworthiness of researchers and the trustworthiness of the institution, more broadly. Less than half of respondents (44.8%; Table 4, Q2) agreed or strongly agreed that the institution communicates the value of community engagement in research across the organization, and only one third (34.2%; Table 4, Q7) agreed or strongly agreed that the institution’s plan for action is co-developed with community members. Across these domains, institutional respondents expressed significantly greater skepticism than community members, echoing qualitative findings that emphasized the need for authentic and co-created engagement beyond grant-driven requirements.

In contrast, perceptions of individual researchers were consistently more favorable. Nearly two-thirds (65.9%; Table 4, Q4) of respondents agreed or strongly agreed that *researchers* support honest communication with the communities and over half (55.3%; Table 4, Q8) agreed or strongly agreed that *researchers* create an open, honest, and accessible environment where community members feel safe and able to provide feedback. These findings underscore the need to take a multifaceted focus on the institution’s reputation in the community, suggesting that institutional reputation is shaped not only by clinical excellence but also by individual clinicians’ and researchers’ commitment and embodiment of the institution’s mission, vision, and value with the way that they conduct themselves inside and outside the institution.

Finally, institution-based respondents were less likely than community-based respondents to perceive institutional leaders’ support for CEnR (17.7% of researchers compared to 39.2% community members; Table 6, Q1) or incorporates community priorities into strategic planning (20.5% of researchers compared to 33.3% community members; Table 6, Q5). Overall, community members perceived stronger institutional support for CEnR than did those working within the institution, underscoring knowledge about persistent internal constraints on prioritizing and sustaining engagement.

**Table 6.** Perceptions of leadership engagement

	Overall		Community		Academic		
Domains/ Statements	*N*	%	*N*	%	*N*	%	*z*-statistic
**1. [Institutional] leadership ensures that researchers have the resources necessary to conduct CEnR**							
Strongly disagree	5	5.9	1	2.0	4	11.8	−2.684 (*p* = 0.007)
Disagree	9	10.6	4	7.8	5	14.7	
Neither agree nor disagree	18	21.2	8	13.7	11	32.4	
Agree	20	23.5	16	31.4	4	11.8	
Strongly agree	6	7.1	4	7.8	2	5.9	
Don’t know	13	15.3	11	21.6	2	5.9	–
Missing	14	16.5	8	15.7	6	17.6	–
**2. [Institutional] leadership strongly supports training and development of CEnR researchers**							
Strongly disagree	5	5.9	2	3.9	3	8.8	−2.557 (*p* = 0.011)
Disagree	10	11.8	3	5.9	7	20.6	
Neither agree nor disagree	17	20.0	8	15.7	9	26.5	
Agree	22	25.9	16	31.4	6	17.6	
Strongly agree	8	9.4	6	11.8	2	5.9	
Don’t know	9	10.6	8	15.7	1	2.9	–
Missing	14	16.5	8	15.7	6	17.6	–
**3. [Institutional] leadership supports researchers to learn from community partners**							
Strongly disagree	4	4.7	1	2.0	3	8.8	−1.677 (*p* = 0.094)
Disagree	9	10.6	5	9.8	4	11.8	
Neither agree nor disagree	12	14.1	5	9.8	7	20.6	
Agree	26	30.6	17	33.3	9	26.5	
Strongly agree	10	11.8	7	13.7	3	8.8	
Don’t know	10	11.8	8	15.7	2	5.9	–
Missing	14	16.5	8	15.7	6	17.6	–
**4. [Institutional] leadership consistently promotes the value of CEnR across the organization**							
Strongly disagree	6	7.1	2	3.9	4	11.8	−2.138 (*p* = 0.032)
Disagree	13	15.3	6	11.8	7	20.6	
Neither agree nor disagree	14	16.5	6	11.8	8	23.5	
Agree	18	21.2	11	21.6	7	20.6	
Strongly agree	5	5.9	5	9.8	0	0	
Don’t know	13	15.3	11	21.6	2	5.9	–
Missing	16	18.8	10	19.6	6	17.6	–
**5. [Institutional] leadership considers community priorities in strategic planning**							
Strongly disagree	8	9.4	2	3.9	6	17.6	−2.453 (*p* = 0.014)
Disagree	11	12.9	5	9.8	6	17.6	
Neither agree nor disagree	13	15.3	6	11.8	7	20.6	
Agree	18	21.2	12	23.5	6	17.6	
Strongly agree	6	7.1	5	9.8	1	2.9	
Don’t know	15	17.6	13	25.5	2	5.9	–
Missing	14	16.5	8	15.7	6	17.6	–

### Integration to generate actionable recommendations

Qualitative and quantitative data were integrated at multiple points during survey development, through comparison of qualitative themes and quantitative patterns and interpretation of findings to generate institutional-level recommendations for institutional leadership, researchers and their staff, and community members. In collaboration with the institutional team, recommendations were grounded in partner experiences with the goal to identify pragmatic and contextually informed strategies to strengthen institutional capacity.

A consolidated recommendation for leaders was to establish and strengthen a clear institutional vision for CEnR through building a “learning health system” across all clinical, educational, and research arms. A concrete recommendation was for the leaders to centralize CEnR by creating “one front door” for communities to engage with investigators. For researchers and their staff, recommendations were to increase recruitment and retention of investigators for CEnR through protected time, bridge and pilot funding, and mentorship. Recommendations for communities were to increase funding for partnerships and for community-based organizations for their navigation staff as well as to support training for community members to lead research activities (see Table 7 for all recommendations).

**Table 7.** Actionable recommendations for supporting community-engaged research at the institution

**For leadership**: Establish a clear institutional vision for CEnR across the clinical, research and education arms From a scientific perspective, leverage the innovative opportunity to address the intersection of learning health systems and pragmatic trials with CEnR Centralize leadership for CEnR to create “one front door” for communities to engage with the institution Generate linkages between community health needs assessments and CEnR researchers Align existing internal resources that address community engagement and health equity Engage the institutional Public Affairs team in establishing a budget in support of CEnR (i.e., projects that go beyond community engagement) Proactively seek to enhance diversity of patients for improved trust and research recruitment Create policies for consistent dissemination of research and use of resources for improving health in the community Support a community steering committee in setting strategic priorities for CEnR Generate criteria for rigorous CEnR that receives internal support, through engagement of external experts and steering committee members who represent the community Create engagement opportunities for research directors across the institution to engage with the steering committee to co-develop CEnR strategic priorities **For supporting researchers and staff**: Increase recruitment of investigators for CEnR Improve retention for CEnR through protected time, direct mentorship from senior investigators and paid community mentors Create fiscal support for researchers engaged in CEnR, including bridge funding (i.e., between periods of NIH funding) to support partnerships through times of inherent volatility Increase institution-wide and disease agnostic, internal/pilot funding opportunities for CEnR or repurpose existing opportunities to include a CEnR component Recognize the need for institutional infrastructure support for CEnR engagement activity and partnership work that falls outside the work of developing and implementing research protocols **For supporting community members**: Increase funding to the community for developing CEnR partnerships for research as a stepwise strategy Subcontract with community-based organizations to support their own community research navigators (as opposed to institutional staff) Support a pipeline of training and further education for community members to lead research activities and become investigators Establish bi-directional mentorship for investigators and community members

## Discussion

This study presents findings from an evaluation of institutional support for CEnR at a multi-site academic medical center, with implications for other large, complex academic health systems. Building on prior scholarship [8–12] that has largely examined CEnR at the partnership levels, this study shifts the analytic focus upstream to examine institutional conditions that shape whether such partnerships can be sustained in academic medical centers [16,18,22]. This mixed-methods study designed to capture perspectives from both community and academics and leaders expands the literature around translational sciences and community engagement infrastructure and describes how academic medical center structures, policies, and practices support CEnR. Guided by the domains from the community-based participatory research model [19] and the National Academies, Assessing Meaningful Community Engagement model [20], this study examined the alignment between the institutional mission, vision, and values; perceptions of the institutional trustworthiness and reputation; operational policies and practices related to CEnR; and leadership investment and prioritization of CEnR.

Overall, survey findings indicate a clear opportunity to bridge the gap between institutional aspirations for community health and the operational realities of CEnR. There is some complexity in which community respondents distinguished between the academic medical center as an institution and the individual researchers with whom they’ve interacted, suggesting that a positive partnership-level experience may coexist with broader institutional constraints. This distinction mirrors prior work highlighting difference between interpersonal trust in researchers and trust in institutions more broadly [8,26], requiring further study. Perspectives from respondents embedded within the institution further underscored the challenge of prioritizing CEnR within large academic medical centers whose dominant orientation remains complex, tertiary, clinical care, and is also noted as a tension in the evaluation of institutional readiness for community engagement across clinical and translational science centers [1].

The qualitative findings deepen and contextualize these survey patterns by illuminating the institutional dynamics through which CEnR is supported. Across five themes, participants described the need for stronger integration between research and clinical care, particularly in response to community members’ concerns about research participation without access to or utilization of clinical services. Conceptualizing CEnR as a part of a learning healthcare system [27], in which community priorities, clinical practice, and research mutually inform one another could be a promising strategy that addresses this disconnect and aligns with the calls in translational science to embed engagement within care delivery rather than treating it as a parallel activity [1,27].

Participants also highlighted how the institution’s global reputation creates both trust in research and clinical excellence while creating perceived distance from local community priorities. This reinforces the importance of sustained, place-based engagement beyond individual projects and is consistent with the prior literature, where community members emphasized the need for institutional commitment to CEnR that is proactive rather than in reaction to grant requirements, alongside intentional efforts to overcoming mistrust, historical tensions, and structural barriers that limit long-term community partnerships [4].

The participatory sense-making, based on CBPR principles, with the institutional team allowed for the translations of these complex contextual insights into actionable, role-specific recommendations targeting institutional leaders, researchers and staff, and community partners. Recommendations for leaders emphasize articulating a shared vision for CEnR across the clinical, educational, and research mission areas and reducing fragmentation through the centralized coordination or a “front door” for engagement. Recommendations for researchers and staff focus on strengthening institutional supports for CEnR through protected time, mentorship and trainings; bridge funding between awards to sustain community partnering processes; and reductions in administrative processes that strain community partnerships. Finally, recommendations for community partners underscore the direct investment in community-based organizations, research leadership development, and bi-directional mentorship to support equitable participation and shared governance. Collectively, these recommendations reinforce that effective CEnR is not solely the responsibility of individual partnerships and requires intentional support across the institution to achieve sustained community impact.

Several limitations should be considered when interpreting these findings. First, most community participants were engaged in advisory boards or existing community-engaged research partnerships and thus may represent individuals with a greater familiarity with research processes and a nuanced understanding of CEnR compared to community members not engaged in research activities. Future studies should intentionally include a broader range of community members, including those without prior research involvement to capture a wider spectrum of experiences with academic medical centers. Second, the survey responses from community members on some questions may reflect specific projects rather than perceptions of the institution as a whole, underscoring the need for further psychometric testing and refinement of institutional-level measures of CEnR support and trustworthiness. We also carefully considered the additional sub group analyses by years of CEnR experience and level of engagement across the CEnR continuum; however, given the modest sample size and the risk of over-stratification, we did not conduct these analyses and instead identify them as important areas for future research. Finally, the cross-sectional nature of assessment limits the ability to examine how institutional conditions and perceptions of support for CEnR evolve over time, particularly in response to organizational change or national context. We attempted to mitigate these limitations through a theory-guided analyses, team-based sense-making, and integration of qualitative and quantitative data for interpretation. This mixed-methods approach provides a robust understanding of contextual conditions shaping CEnR and informed the development of recommendations for how to improve structures, policies, and practices to enable institutions to realize their vision of supporting and growing their footprint in innovative CEnR.

Concomitant with the evaluation process and presently, evaluation findings are being used by community and academic partners to enhance and influence the institutional conditions to promote CEnR and health equity research. For example, a CEnR “playbook” of best practices for CEnR at the institutional level has been developed for community and academic audiences, with the dual objectives to outline best practices for CEnR and to shed light on institutional facilitators that are lacking or should be revised (e.g., around remuneration processes for community partners, IRB nuances for CEnR). The evaluation also led to a change in intramural CEnR grant-making processes to extend funding intervals for partnership development and funding amounts. The evaluation has also informed a CEnR strategic plan at the institutional level to promote health equity.

Findings from this study suggest that a systematic process of prioritizing the perspectives of academic leaders, researchers, community engagement staff, and community members can provide context-specific recommendations to support and promote CEnR at an institutional level. Actions on these recommendations can facilitate CEnR partnerships to promote health equity across the clinical translational science centers.

## Supporting information

10.1017/cts.2026.10771.sm001 Adsul et al. supplementary material Adsul et al. supplementary material

## Supplementary material

The supplementary material for this article can be found at https://doi.org/10.1017/cts.2026.10771.
