# Supplementary material for: What does it take to support community-engaged research in an academic medical center? A mixed-methods study of community and academic perspectives
Source: J Clin Transl Sci. 2026 Jul 16;10(1):e123. doi: 10.1017/cts.2026.10771 (PMC13420142; doi:10.1017/cts.2026.10771)
Supplement: Adsul et al. supplementary material [file S2059866126107717sup001.docx]

**Good Reporting of a Mixed Methods Study (GRAMMS) checklist**

| **Guidelines** | **How guidelines were addressed?** | **Location in the manuscript** |
| --- | --- | --- |
| **Justification:** Describe why a mixed methods approach was used to answer the research question. | We justify the use of mixed methods to comprehensively assess institutional conditions supporting CEnR, noting that quantitative data alone cannot capture contextual and relations dynamics, while qualitative data alone cannot assess the distribution of perceptions across the groups. | Introduction and Study design |
| **Design Details:** Explain the mixed methods design, including the purpose, priority (which method is primary), and sequence of methods (e.g., concurrent, sequential). | The study is described as a sequential exploratory mixed methods design, with qualitative interviews and focus groups conducted first, followed by a quantitative survey informed by qualitative findings. Qualitative and quantitative components informed the participatory and iterative interpretation of data for actionable recommendations | Study design, Data Analyses |
| **Component Reporting:** Detail sampling, data collection, and analysis for both quantitative and qualitative parts separately. | Separate sections describe the recruitment, collection and analyses across the respondent groups. | Methods |
| **Integration:** Explain where, how, and who participated in integrating the quantitative and qualitative data. | Integration is described at multiple points: (1) during survey development informed by qualitative findings; (2) through comparison of qualitative themes and quantitative patterns, and (3) during interpretation to generate institutional-level recommendations. Participatory sense-making discussions involved academic and community-engaged investigators. | Methods, Data analyses; Results (integration paragraph) |
| **Limitations:** Describe any limitations of one method that arose because of the other method. | We acknowledge the constraints related to sample composition, interpretation of institutional level perceptions, modest survey sample size, and analytic tradeoff for a cross sectional study. The manuscript explains how integration across methods mitigated limitations of any single approach. | Discussion, paragraph 4, limitations |
| **Insights:** Report any unique insights or findings that came specifically from mixing or integrating the methods. | The manuscript explicitly states that integration enabled the identification of academic and community perspectives and supported the translation of contextual findings into actionable, role-specific recommendations. | Results, Discussion |

Citation: O'Cathain A, Murphy E, Nicholl J. The quality of mixed methods studies in health services research. J Health Serv Res Policy. 2008;13(2):92-98
